# Identification of alternative topological domains in chromatin

**DOI:** 10.1186/1748-7188-9-14

**Published:** 2014-05-03

**Authors:** Darya Filippova, Rob Patro, Geet Duggal, Carl Kingsford

**Affiliations:** 1 Lane Center for Computational Biology, Carnegie Mellon University, Pittsburgh PA, USA; 2 Joint Carnegie Mellon University — University of Pittsburgh Ph.D. Program in Computational Biology, Pittsburgh PA, USA

**Keywords:** Alternative topological domains, Chromatin conformation capture, Dynamic programming

## Abstract

Chromosome conformation capture experiments have led to the discovery of dense, contiguous, megabase-sized topological domains that are similar across cell types and conserved across species. These domains are strongly correlated with a number of chromatin markers and have since been included in a number of analyses. However, functionally-relevant domains may exist at multiple length scales. We introduce a new and efficient algorithm that is able to capture persistent domains across various resolutions by adjusting a single scale parameter. The ensemble of domains we identify allows us to quantify the degree to which the domain structure is hierarchical as opposed to overlapping, and our analysis reveals a pronounced hierarchical structure in which larger stable domains tend to completely contain smaller domains. The identified novel domains are substantially different from domains reported previously and are highly enriched for insulating factor CTCF binding and histone marks at the boundaries.

## Background

Chromatin interactions obtained from a variety of recent experimental techniques in chromosome conformation capture (3C) [1] have significantly advanced our understanding of the geometry of chromatin structure [2], its relation to the regulation of gene expression, nuclear organization, cancer translocations [3], and copy number alterations in cancer [4]. Recently, dense, contiguous regions of chromatin termed *topological domains* have been discovered in both mammals [5] and in fruit flies [6]. Topological domains have since been incorporated into many subsequent analyses [7-9] due to the fact that they are persistent across cell types, conserved across species, and serve as a skeleton for the placement of many functional elements of the genome [10,11].

**Figure 1:**
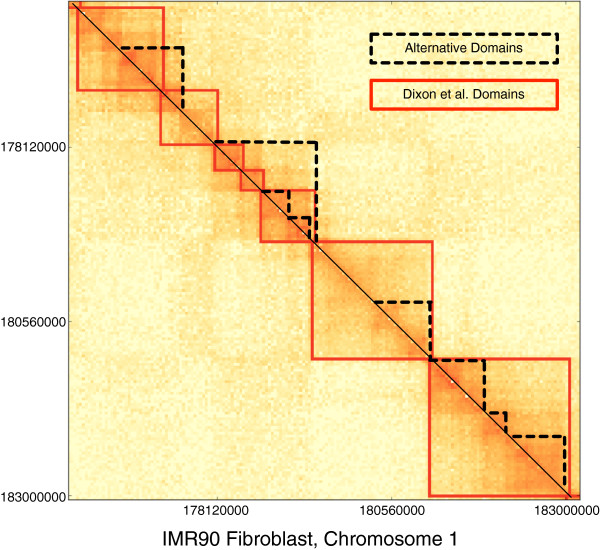
**Interaction matrix for a portion of human chromosome 1 from a recent Hi-C experiment by Dixon et al. **[5]**.** Each axis represents a location on the chromosome with a step of 40kbp. Densely interacting domains identified by the method of Dixon et al. are shown in red. Alternative domains are shown as dotted black lines on the upper triangular portion of the matrix. Visual inspection of the lower triangular portion suggests domains could be completely nested within another and highly overlapping when compared to Dixon et al.’s domains. This motivates the problem of identifying alternative domains across length scales.

3C experiments result in matrices of counts that represent the frequency of cross-linking between restriction fragments of DNA that are spatially near one another. The identification of domains in Dixon et al. [5] employed a Hidden Markov Model (HMM) on these interaction matrices to identify regions initiated by significant downstream chromatin interactions and terminated by a sequence of significant upstream interactions. A defining characteristic of the domains from their analysis is that higher frequency 3C interactions tend to occur within domains as opposed to across domains. This aspect of domains is also reflected in the block-diagonal structure of 3C interaction matrices as shown in Figure 1. In this sense, domains can be interpreted as contiguous genomic regions that self-interact frequently and are more spatially compact than their surrounding regions.

However, the single collection of megabase-sized domains may not be the only topologically and functionally relevant collection of domains. On closer inspection of the block-diagonal matrix structure in Figure 1, it becomes clear that there are alternative contiguous regions of the chromosome that self-interact frequently and are likely more spatially compact than their surrounding regions (dotted lines). Some of these regions appear to be completely nested within others, suggesting a hierarchy of compact regions along the chromosome, while others appear to overlap each other. These observations suggest that functionally-relevant chromosomal domains may exist at multiple scales potentially contributing to a hierarchy of domains or a more complex relationship between domains.

We introduce a new algorithm to efficiently identify topological domains in 3C interaction matrices for a given domain-length scaling factor *γ*. Our formulation of this problem as a dynamic program allows for an efficient traversal of the solution space to obtain alternative optimal and near-optimal domain sets. Our results suggest that there exist a handful of characteristic resolutions across which domains are similar. Based on this finding, we identify a consensus set of domains that persists across various resolutions. We find that domains discovered by our algorithm are dense and cover interactions of higher frequency than inter-domain interactions. Additionally, we show that inter-domain regions within the consensus domain set are highly enriched with insulator factor CTCF and histone modification marks. We analyze a set of domains from multiple optimal domain sets across scales and establish that the organization of domains is highly hierarchical, suggesting that the generated domains can be used as the basis for understanding the hierarchical organization of the genome and its role in gene regulation. We argue that our straightforward approach retains the essence of the more complex multi-parameter HMM introduced in [5] while allowing for the flexibility to identify biologically relevant domain structures at various scales.

## Problem definition

Given the resolution of the 3C experiment (say, 40kbp), the chromosome is broken into *n* evenly sized fragments. 3C contact maps record interactions between different sections of the chromosome in the form of a weighted adjacency matrix **A** where two fragments *i* and *j* interact with frequency **A**_*i* *j*_.

### **Problem1** (Resolution-specific domains).

Given a *n*×*n* weighted adjacency matrix **A** and a resolution parameter *γ*≥0, we wish to identify a set of domains *D*_*γ*_ where each domain is represented as an interval *d*_*i*_= [ *a*_*i*_,*b*_*i*_], 1≤*a*_*i*_<*b*_*i*_≤*n* such that no two *d*_*i*_ and *d*_*j*_ overlap for any *i*≠*j*. Additionally, each domain should have a larger interaction frequency within the domain than to its surrounding regions.

Specifically, we seek to identify a set of non-overlapping domains *D*_*γ*_ that optimizes the following objective:

(1) $$\max\underset{[a_{i},b_{i}]\in D_{\gamma }}{\sum }q(a_{i},b_{i},\gamma ),$$

where *D*_*γ*_ chosen from the set of all possible domains, and *q* is a function that quantifies the quality of a domain [*a*_*i*_,*b*_*i*_] at resolution *γ*. Here, the parameter *γ* is inversely related to the average domain size in *D*_*γ*_: lower *γ* results in sets of larger domains and higher *γ* corresponds to sets of smaller domains. Since domains are required to contain consecutive fragments of the chromosome, this problem differs from the problem of clustering the graph of 3C interactions induced by **A**, since such a clustering may place non-contiguous fragments of the chromosome into a single cluster. In fact, this additional requirement allows for an efficient optimal algorithm.

### **Problem2** (Consensus domains across resolutions).

Given **A** and a set of resolutions *Γ*={*γ*_1_,*γ*_2_,…}, identify a set of non-overlapping domains *D*_*c*_ that are most persistent across resolutions in *Γ*:

(2) $$\max\underset{[a_{i},b_{i}]\in D_{c}}{\sum }p(a_{i},b_{i},\Gamma ),$$

where *D*_*c*_ is the set of non-overlapping persistent domains across resolutions, and *p*(*a*_*i*_,*b*_*i*_,*Γ*) is the persistence of domain [ *a*_*i*_,*b*_*i*_] corresponding to how often it appears across resolutions.

## Algorithms

### Domain identification at a particular resolution

Since each row and corresponding column in a 3C interaction matrix encodes a genomic position on the chromosome, we can write the solution to objective (1) as a dynamic program:

(3) $${\text{OPT}}_{1}(l)=\underset{k<l}{\max}\{{\text{OPT}}_{1}(k-1)+\max\{q(k,l,\gamma ),0\}\},$$

where OPT_1_(*l*) is the optimal solution for objective (1) for the sub-matrix defined by the first *l* positions on the chromosome (OPT_1_(0)=0). The choice of *k* encodes the size of the domain immediately preceding location *l*. We define negative-scoring domains as non-domains and, as such, only domains with *q*>0 in the max term in (3) are retained.

Our quality function *q* is:

(4) $$q(k,l,\gamma )=s(k,l,\gamma )-\mu_{s}(l-k),$$

where

(5) $$s(k,l,\gamma )=\frac{\sum_{g=k}^{l}\sum_{h=g+1}^{l}A_{\text{gh}}}{{\left(l-k\right)}^{\gamma }}$$

is a *scaled density* of the subgraph induced by the interactions *A*_*g* *h*_ between genomic loci *k* and *l*. When *γ*=1, the scaled density is the weighted subgraph density [12] for the subgraph induced by the fragments between *k* and *l*, which is the upper-triangular portion of the submatrix defined by the domain in the interval [ *k*,*l*] divided by the scaled length (*l*−*k*)^*γ*^ of the domain. When *γ*=2, the scaled density is half the internal density of a graph cluster [13]. For larger values of *γ*, the length of a domain in the denominator is amplified, hence, smaller domains would produce larger objective values than bigger domains with similar interaction frequencies. Equation (4) is the zero-centered sum of (5). *μ*_*s*_(*l*−*k*) is the mean value of (5) over all sub-matrices of length *l*−*k* along the diagonal of **A**, and can be pre-computed for a given **A**. We disallow domains where there are fewer than 100 sub-matrices available to compute the mean. By doing this, we are only excluding domains of size larger than *n*−100 fragments, which in practice means that we are disallowing domains that are hundreds of megabases long. Values for the numerator in (5) are also pre-computed using an efficient algorithm [14], resulting in an overall run-time of *O*(*n*^2^) to compute OPT_1_(*n*).

### Enumerating multiple optimal and near-optimal solutions

The set of domains found by the dynamic program in Equation 3 may not be the only set obtaining the maximum value of OPT_1_(·). In fact, there may be multiple optimal solutions and solutions which are near optimal. The domain structures that appear in alternative optimal or near optimal solutions are of interest, especially if they are significantly different, since they represent a potentially diverse array of alternative domains that are only precluded from the initially computed optimal solution as a result of the arbitrary breaking of ties that takes place in the dynamic program. We wish to be able to account for such alternative solutions by enumerating them efficiently and in order of a decreasing solution score.

Since Equation 3 will allow ‘non-domains’ (i.e. intervals on the chromosome with *q*(*k*,*l*,*γ*)≤0) to be split arbitrarily without affecting the optimal score, we modified the procedure as shown in Equation 6 to explicitly disallow adjacent non-domains:

(6) $${\text{OPT}}_{1}^{\prime }(l)=\max\;\left\{\begin{array}{l} \underset{k<l}{\max}\{{\text{OPT}}_{\text{D}}(k-1)\}\\ {\text{OPT}}_{\text{D}}(l), \end{array}\right)$$

where the optimal score of *l* ending a domain is

(7) $${\text{OPT}}_{\text{D}}(l)=\underset{k<l}{\max}\{{\text{OPT}}_{1}^{\prime }(k-1)+q^{\prime }(k,l,\gamma )\},$$

and the quality function for the domain is

(8) $$q^{\prime }(k,l,\gamma )=\left\{\begin{array}{l} q(k,l,\gamma )\;\text{if}\;\;q(k,l,\gamma )>0\\ -\infty \;\;\;\text{otherwise.} \end{array}\right)$$

${\text{OPT}}_{\text{D}}(l)={\text{OPT}}_{1}^{\prime }(l)=0$ for *l* ∈ {0,1}. In Equation 6, max*k*<*l*OPT_D_(*k*−1) represents the optimal score at *l* where *l* ends a non-domain region. This solution to Problem 1 produces a set of domains with the same optimal score as Equation 3, but guarantees that alternative optimal and near-optimal domain sets do not contain non-domains that are adjacent.

To efficiently identify alternative optimal and near-optimal solutions, we use the fact that the dynamic program in Equation (6) can be conceptually represented as a directed acyclic graph  where each ${\text{OPT}}_{1}^{\prime }(l)$ and OPT_D_(*l*) is connected by an edge to every other term it depends on: ${\left\{{\text{OPT}}_{1}^{\prime }(k)\right\}}_{k<l}$ and {OPT_D_(*k*)}_*k*<*l*_. For each edge *e*=(*k*,*l*) in , the weight of *e* is *q*^′^(*k*,*l*,*γ*). Thus, finding a set of domains with an optimal score is equivalent to finding a highest-weight path in  starting from the node representing ${\text{OPT}}_{1}^{\prime }(n)$. To find the top-*K* solutions, we then find the *K* highest weight paths in  using a standard procedure [15].

### Obtaining a consensus set of persistent domains across resolutions

For objective (2), we use the procedure above to construct a set $D=\underset{\gamma \in \Gamma }{⋃}D_{\gamma }$.  is a set of overlapping intervals or domains, each with a quality score defined by its persistence *p* across resolutions. To extract a set of highly persistent, non-overlapping domains from , we reduce problem 2 to the weighted interval scheduling problem [16], where competing requests to reserve a resource in time are resolved by finding the highest-priority set of non-conflicting requests. To find a consensus set of domains, we map a request associated with an interval of time to a domain and its corresponding interval on the chromosome. The priority of a request maps to a domain’s persistence *p* across length scales.

The algorithm to solve problem 2 is then:

(9) $$\begin{array}{l} {\text{OPT}}_{2}(\;j)=\max\{{\text{OPT}}_{2}(j-1),\\ \;{\text{OPT}}_{2}(c(\;j))+p(a_{j},b_{j},\Gamma )\} \end{array}$$

where OPT_2_(*j*) is the optimal non-overlapping set of domains for the *j*th domain in a list of domains sorted by their endpoints (OPT_2_(0)=0), and *c*(*j*) is the closest domain before *j* that does not overlap with *j*. The first and second terms in (9) correspond to either choosing or not choosing domain *j* respectively. We pre-compute a domain’s persistence *p* as:

(10) $$p(a_{i},b_{i},\Gamma )=\underset{\gamma \in \Gamma }{\sum }\delta_{i}\;\;\text{where}\;\;\delta_{i}=\left\{\begin{array}{l} 1\;\text{if}\;\;[\;a_{i},b_{i}]\in D_{\gamma }\\ 0\;\text{otherwise.} \end{array}\right)$$

Equation (10) is therefore a count of how often domain *i* appears across all resolutions in *Γ* for domain sets identified by the dynamic program at a single resolution. It may be desirable to treat multiple highly overlapping, non-equivalent domains as a single domain, however, we conservatively identify exact repetitions of a domain across resolutions since this setting serves as a lower bound on the persistence of the domain. If m=|D|, then pre-computing persistence takes *O*(*m*|*Γ*|) time, and *c*(*j*) is precomputed after sorting the intervals by their endpoints. The limiting factor when computing OPT_2_(*m*) is the time to compute *c*(*j*), which is of order *m* log*m*. Thus, the overall algorithm runs in *O*(*m* log*m*+(*n*^2^+*m*)|*Γ*|) time taking into account an additional *O*(*n*^2^|*Γ*|) time for computing .

## Results

We used chromatin conformation capture data from Dixon et al. [5] for human fibroblast and mouse embryonic cells. The 3C contact matrices were already aggregated at fragment size 40kb and were corrected for experimental bias according to [17]. We compared our multiscale domains and consensus sets against the domains generated by Dixon et al. for the corresponding cell type and species. For human fibroblast cells, we used CTCF binding sites from [18]. For mouse embryonic cell CTCF binding sites and chromatin modification marks, we used data by Shen et al. [19].

### Ability to identify densely interacting domains across scales

**Figure 2:**
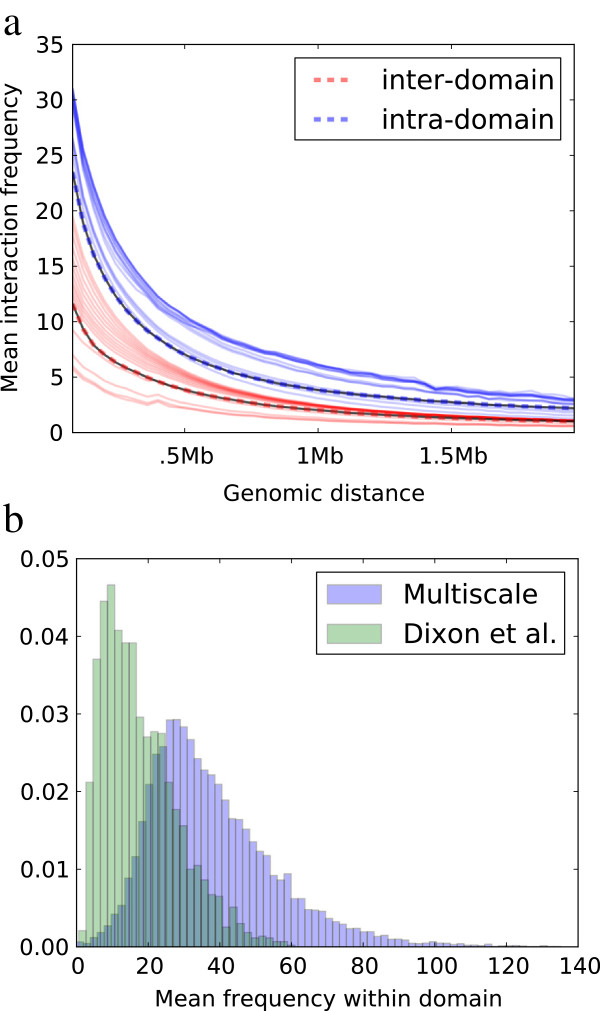
**Our approach identifies densely interacting domains across scales.(a)** Our algorithm discovers domains with mean frequency value for inter- and intra-domain interactions (solid lines) at or better than that of Dixon et al. domains (dotted lines). Each solid line represents domains at different resolution *γ* in human fibroblast cells. **(b)** Multiscale domains identified in human fibroblast cells by our dynamic program tend to have higher mean frequency than those of Dixon et al. (distributions are plotted after outliers >*μ*+4*σ* were removed).

Multiresolution domains successfully capture high frequency interactions and leave interactions of lower mean frequency outside of the domains. We compute the mean interaction frequency for all intra- and inter-domain interactions at various genomic lengths and plot the distribution of means for multiple resolutions (Figure 2(a)). The mean intra-domain interaction frequency (blue) is consistently higher (up to two times) than the mean frequency for interactions that cross domains (red). Compared to the domains reported by Dixon et al., our domains tend to aggregate interactions of higher mean frequency, especially at larger *γ*. The distribution of mean intra-domain frequencies for Dixon et al. is skewed more to the left than that of the multiscale domains (Figure 2(b)). This difference can be partially explained by the fact that multiscale domains on average are smaller in size (*μ*=0.2Mb, *σ*=1.2Mb) than domains reported by Dixon et al. (*μ*=1.2Mb, *σ*=0.9Mb).

### Domain persistence across scales

**Figure 3:**
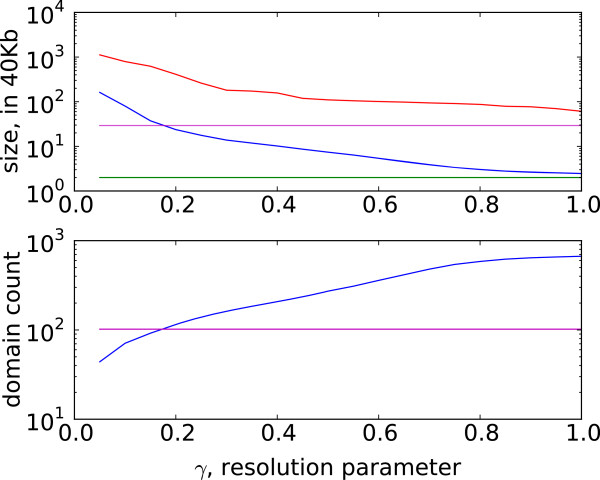
**Domain sizes and count across resolutions.** The domain sizes increase and the domain count decreases as the resolution parameter drops. Above: plotted are maximum (red), average (blue), and minimum (green) domain size averaged over all chromosomes for the domains on human fibroblast cells (IMR90). The magenta line shows the average domain size for domains reported by Dixon et al. Below: the number of domains increases for higher values of resolution parameter. The magenta line displays domain count for Dixon et al.

**Figure 4:**
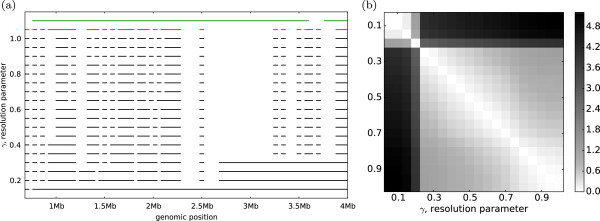
**Domain persistence across scales.(a)** Domains identified by our algorithm (black) are smaller at higher resolutions and merge to form larger domains at ***γ*** close to 0. Visual inspection shows qualitative differences between consensus domains (red) and domains reported by Dixon et al. (green). Data shown for the first 4Mb of chromosome 1. **(b)** Variation of information for domains identified by our algorithm across different resolutions for chromosome 1 in human fibroblast cells.

Domain sets across resolutions share significant similarities, even as the distribution of domains and their sizes begin to change (Figure 3). The patterns of similarity are particularly obvious if we plot the domains at various resolutions (Figure 4(a)): many domains identified by our algorithm persist at several resolutions and are aggregated into larger domains at smaller *γ*, suggesting a hierarchical domain structure. The stability of these domains across resolutions indicates that the underlying chromosomal structure is dense within these domains and that these domains interact with the rest of the chromosome at a much lower frequency.

A pairwise comparison of domain configurations displays regions of stability across multiple resolutions (Figure 4(b)). We use the variation of information (VI) [20], a metric for comparing two sets of clusters, to compute the distance between two sets of domains. To capture the similarities between two domain sets *D* and *D*^′^ and the inter-domain regions induced by the domains, we construct new derivate sets *C* and *C*^′^ where *C* contains all domains *d*∈*D* as well as non-domain regions (*C*^′^ is computed similarly). To compute entropy $H(C)=\sum_{c_{i}\in C}p_{i}\log p_{i}$, we define the probability of seeing each interval *c*_*i*_= [ *a*_*i*_,*b*_*i*_] in *C* as *p*_*i*_=(*b*_*i*_−*a*_*i*_)/*L* where *L* is the length of the chromosome. When computing the mutual information $I(C,C^{\prime })=\sum_{c_{i}\in C}\sum_{c_{j}^{\prime }\in C^{\prime }}p_{\text{ij}}\log[\;p_{\text{ij}}/(p_{i}p_{j})]$ between two sets of intervals *C* and *C*^′^, we define the joint probability *p*_*i* *j*_ to be |[ *a*_*i*_,*b*_*i*_]∩[ *a*_*j*_,*b*_*j*_]|/*L*.

We then compute variation of information on these two new sets: *VI*(*C*,*C*^′^)=*H*(*C*)+*H*(*C*^′^)−2*I*(*C*,*C*^′^). Chromosome 1, for example, has three visually pronounced groups of resolutions within which domain sets tend to be more similar than across (*γ*= [0.00-0.20], [0.25-0.70], and [0.75-1.00] — see Figure 4(b)).

### Comparison with the previously identified set of domains in Dixon et al

At higher resolutions, domains identified by our algorithm are smaller than those reported by Dixon et al. (Figure 3). As the resolution parameter decreases to 0.0, the average size of the domains increases. The composition of the domains we identify is different from that of Dixon et al. as illustrated in Figure 4(a) and captured by the variation of information in Figure 4(b).

We use the consensus domains algorithm to obtain a consensus set of domains *D*_*c*_ persistent across resolutions. We construct the set *Γ* by defining the range of our scale parameter to be [0,*γ*_max_] and incrementing *γ* in steps of 0.05. In order to more directly compare with previous results, we set *γ*_max_=0.5 for human and 0.25 for mouse since these are the scales at which the maximum domain sizes in Dixon et al.’s sets match the maximum domain sizes in our sets.

**Figure 5:**
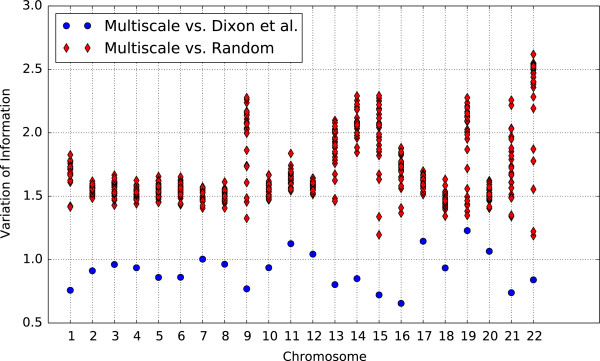
**Comparison of Dixon et al.’s domain set with the multiscale consensus set for chromosomes 1–22 (*x*-axis).** We used the variation of information (VI) (*y*-axis) to compute distances between domain sets for the multiscale consensus set vs. Dixon et al. (blue dots) and the multiscale consensus vs. randomly shuffled domains (red diamonds).

Our consensus domain set agrees with the Dixon et al. domains better than with a randomized set of domains adhering to the same domain and non-domain length distributions (Figure 5 and [21]). Comparing to a set of random domains also helps to verify that our observations are due to the observed sequence of domains and not the distribution of domain lengths. To shuffle Dixon’s domains, we record the length of every domain and non-domain region, and then shuffle these lengths to obtain a randomized order of domains and non-domains across the chromosome. The fact that variation of information is lower between consensus domains and domains reported by Dixon et al. demonstrates that, though the approaches find substantially different sets of topological domains, they still agree significantly more than one would expect by chance.

### Enrichment of CTCF and histone modifications near boundaries

We assess the enrichment of transcription factor CTCF and histone modifications H3K4me3 and H3K27AC within the inter-domain regions induced by the consensus domains. These enrichments provide evidence that the boundary regions between topological domains correlate with genomic regions that act as insulators and barriers, suggesting that the topological domains may play a role in controlling transcription in mammalian genomes [5].

**Figure 6:**
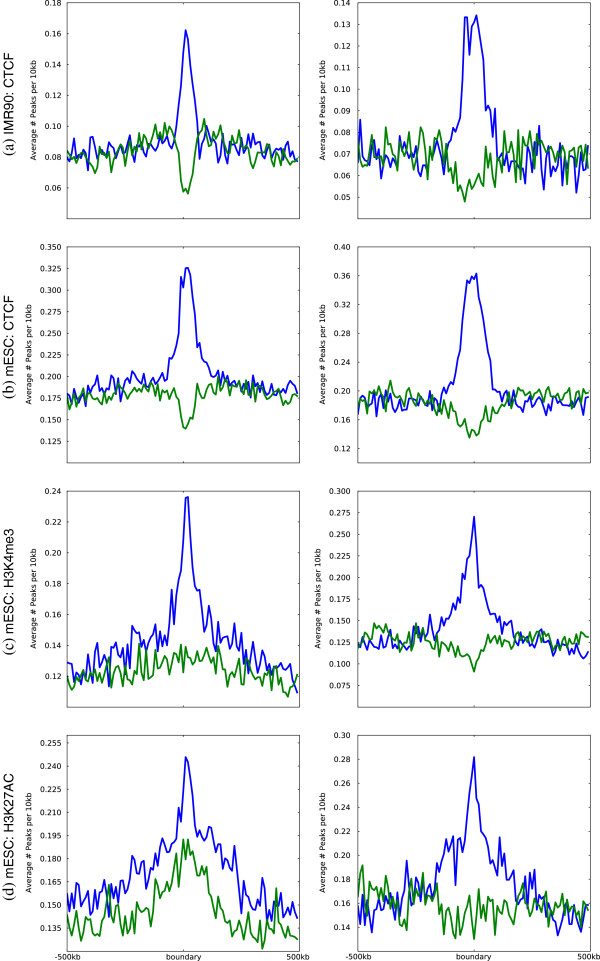
**Enrichment for chromatin marks and histone modifications in domain boundaries.** Enrichment of CTCF binding **(a)** in IMR90 and **(b)** in mESC and histone modifications **(c)**, **(d)** in mESC around domain boundaries for our consensus set of persistent domains (left, blue), and for those identified by Dixon et al. (right, blue). Green lines represent the presence of CTCF at the midpoint of the topological domains.

Figure 6 illustrates the enrichment of insulator or barrier-like elements in domain boundaries in both the human fibroblast (IMR90) and mouse embryonic stem cell (mESC) lines. Specifically, we observe that the boundaries between consensus domains are significantly enriched for all of the transcription factors and histone marks we consider. In certain cases — specifically in the case of CTCF — we notice that the CTCF binding signals peak more sharply in the boundaries between the domains we discover than in the boundaries between the domains of Dixon et al.

We also observe that, when compared with the domain boundaries predicted by Dixon et al., our boundaries more often contain insulator or barrier-like elements (see Table 1). Specifically, we normalize for the fact that we identify approximately twice as many domains as Dixon et al., and generally observe a two-fold enrichment in the fraction of boundaries containing peaks for CTCF markers. This suggests that structural boundaries identified by our method are more closely tied to functional sites which serve as barriers to long-range regulation. We also observe a depletion of insulator CTCF elements within our domains when compared to the domains of Dixon et al. This observation is consistent with the assumption that transcriptional regulation is more active within spatially proximate domains since there are fewer elements blocking regulation within these domains. Table 1 also shows similar patterns for histone modifications which suggests that our domain boundaries are enriched for functional markers of gene regulation.

**Table 1:** Chromatin marks and histone modification enrichments within and between domains

**Signal**	**Domains (**[5]**)**	**Domains (Here)**	**Boundaries (**[5]**)**	**Boundaries (Here)**
CTCF (IMR90)	$\frac{2050}{2234}\approx 0.92$	$\frac{3092}{5365}\approx 0.58$	$\frac{423}{2136}\approx 0.20$	$\frac{2126}{4861}\approx 0.44$
CTCF (mESC)	$\frac{2057}{2066}\approx 1.00$	$\frac{2500}{3578}\approx 0.70$	$\frac{654}{2006}\approx 0.33$	$\frac{2258}{3122}\approx 0.72$
H3K4me3 (mESC)	$\frac{2019}{2066}\approx 0.98$	$\frac{2362}{3578}\approx 0.66$	$\frac{600}{2006}\approx 0.30$	$\frac{1738}{3122}\approx 0.60$
H3K27AC (mESC)	$\frac{1922}{2066}\approx 0.93$	$\frac{2254}{3578}\approx 0.63$	$\frac{458}{2006}\approx 0.23$	$\frac{1342}{3122}\approx 0.43$

Each table entry is of the form $\frac{e}{t}\approx r$ where ***e*** is the number of elements containing ***≥1*** of CTCF and histone modifications, ***t*** is the total number of elements and ***r*** is the approximate ratio ***e/t***. Our method produces more domains, and hence more boundaries, than that of Dixon et al. [5]. However, relative to Dixon et al., our domains are depleted for peaks of interest, while our boundaries are significantly enriched for such peaks.

### Multiple optimal solutions across scales reveal the hierarchical organization of topological domains

**Figure 7:**
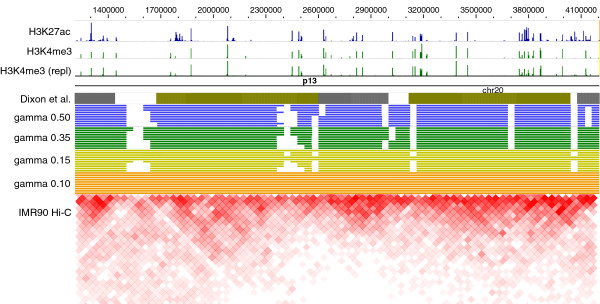
**Domain sets at various resolutions.** 10 best optimal and near-optimal solutions for resolutions *γ*=0.5,0.35,0.15,0.10 for a portion of human fibroblast chromosome 20 (IMR90). Variations in the domain assignments within a single *γ* and across resolutions correspond with visually identifiable, hierarchical regions of dense Hi-C interactions. All histone mark tracks were obtained from IMR90 cells. Plotted with WashU EpiGenome Browser [23].

It has been recently hypothesized that chromatin is packed into the nucleus in a hierarchical manner suggesting that smaller, spatially compact domains combine to form larger superdomains that may be functionally similar [2,3,6]. This hypothesis is partially motivated by the fact that the distribution of 3C interaction frequencies better matches a fractal globule model of chromatin organization than an essentially random equilibrium organization of chromatin in the nucleus [22] and by an initial exploration of the hierarchical organization of the Drosophilla genome [6]. By combining alternative optimal and near-optimal domains across scales, we quantitatively determine the extent to which domains at different *γ* conform to a hierarchical structure empirically identifiable in Figures 4(a) and 7.

We determine the extent to which all identified optimal and near-optimal topological domains are hierarchically organized by combining alternative optimal and near-optimal domains and computing a score characterizing the hierarchy. Specifically, we combine all near-optimal domains across all resolutions into a single set: $D^{K}=\underset{\gamma \in \Gamma }{⋃}\underset{i\in \;[\;1,K]}{⋃}D_{\gamma }^{i}$ where $D_{\gamma }^{i}$ is the *i*th optimal solution at resolution *γ* and *K* total solutions are found at each resolution. We quantify the extent to which domains in this set are nested by determining the fraction of sufficiently different domain pairs {*d*_*i*_,*d*_*j*_} where either *d*_*i*_ or *d*_*j*_ is completely contained in the other:

(11) $$h(D_{\alpha }^{K})=\frac{1}{\left|D_{\alpha }^{K}\right|}\underset{\{d_{i},d_{j}\}\in D_{\alpha }^{K}}{\sum }\delta (d_{i},d_{j}),$$

(12) $$\delta (d_{i},d_{j})=\left\{\begin{array}{l} 1,\;\text{if}\;\;d_{i}\subset d_{j}\;\;\text{or}\;\;d_{j}\subset d_{i}\\ 0,\;\text{otherwise,} \end{array}\right)$$

and $D_{\alpha }^{K}$ contains all pairs of domains {*d*_*i*_,*d*_*j*_} from domains in $D^{K}$ such that *α*=|*d*_*i*_*Δd*_*j*_|/|*d*_*i*_∪*d*_*j*_| — a fraction of genomic fragments different between two domains *d*_*i*_ and *d*_*j*_ in relation to the union of all fragments comprising the two domains — is greater than a user-specified value. For our tests, we define two domains to be different if more than 10% of their fragments differ (*α*=0.1). If no domain is contained fully in any other domain the score *h*(·)=0. If, for every pair of domains, one of the domains is fully contained in the other, the score attains its maximum value *h*(·)=1. We empirically observe that randomly generated domains result in *h*(·)≈0.5.

To determine whether the set of all identified domains we observe is significantly more hierarchical than expected by chance, we randomly shuffle domains while maintaining the same domain and non-domain length distributions as the sets of domains we find [21]. At each resolution, we identify the *K*=10 optimal and near-optimal solutions for all chromosomes in human fibroblast cell line (IMR90) as well as mouse embryonic cells (mESC). The choice of *K*=10 is computationally beneficial given that even for such low *K*, the score for the next optimal solution drops off fast at lower *γ*, but for *γ*=0.5 the optimal score only changes by 0.02% (from 16774.7 to 16771.2) after 50000 solutions are considered. Alternatively, a weaker null hypothesis could be constructed that uses randomly shuffled Hi-C matrix. However, this approach does not control for the distribution of domain lengths — a previously established property of topological domains [5,6]. In addition, it has recently been shown that randomly shuffled Hi-C matrices lack a clear domain structure since they exhibit significantly depleted insulation scores [24]. This weaker null hypothesis is thus not appropriate for determining the significance of hierarchical domain structure. For both organisms, we find that *h*(·) for the identified set of domains is significantly larger than *h*(·) for the randomized domains (Benjamini-Hochberg corrected *P*<0.001 over all chromosomes). The mean value of the identified set of domains is ≈0.95 as opposed to ≈0.70 for 1,000 randomized sets of domains sampled from each resolution. Computing *h*(·) on the combined set of domains is conservative since it is likely that domains from multiple optimal and near-optimal solutions can overlap but may not be completely contained in one another within a length scale. This suggests that the multiple optimal and near-optimal domains across scales exhibit a hierarchical structure and that the ensemble of domains can be used as the basis of a more detailed analysis of the hierarchical organization of these genomes.

## Discussion and conclusions

In this paper, we introduce an algorithm to identify topological domains in chromatin using interaction matrices from recent high-throughput chromosome conformation capture experiments. Our algorithm produces domains that display much higher interaction frequencies within the domains than in-between domains (Figure 2) and for which the boundaries between these domains exhibit substantial enrichment for several insulator and barrier-like elements (Figure 6). To identify these domains, we use a multiscale approach that finds domains at various size scales and generates multiple optimal and near-optimal solutions.

We define a consensus set to be a set of domains that persist across multiple resolutions and give an efficient algorithm that finds such a set optimally.

Our method uses a score function that encodes the quality of putative domains in an intuitive manner based on their local density of interactions. Variations of the scoring function in (4), for example, by median centering rather than mean centering or by optimizing the homogeneity of interaction frequencies instead of total frequencies, can be explored to test the robustness of the enrichments described here.

Our method is particularly appealing in that it requires only a single user-specified parameter *γ*_max_. For our experiments, the parameter *γ*_max_ was set based on the maximum domain sizes observed in Dixon et al.’s experiments so that we could easily compare our domains to theirs. This parameter can also be set intrinsically from properties of the Hi-C interaction matrices. For example, we observe similar enrichments in both human and mouse when we set *γ*_max_ to be the smallest *γ*∈*Γ* such that the median domain size is >80kbp (two consecutive Hi-C fragments at a resolution of 40kbp). This is a reasonable assumption since domains consisting of just one or two fragments do not capture higher-order spatial relationships (e.g. triad closure) and interaction frequencies between adjacent fragments are likely large by chance [22].

We compared the fraction of the genome covered by domains identified by Dixon et al. vs. the domains obtained from our method at various resolutions. Dixon et al.’s domains cover 85% of the genome while our sets tend to cover less of the genome (≈ 65% for a resolution that results in the same number of domains as those of Dixon et al.). The fact that our domain boundaries are more enriched for CTCF sites indicates that our smaller, more dense domains may be more desirable from the perspective of genome function. The dense, functionally-enriched domains discovered by our algorithm provide strong evidence that alternative chromatin domains exist and that a single length scale is insufficient to capture the hierarchical and overlapping domain structure visible in heat maps of 3C interaction matrices.

We provided the first quantitative analysis testing the hypothesis that the domain structure across scales is significantly hierarchically organized, suggesting that the domains we identify can be used as the basis for studying the hierarchical organization of genomes and how this structure impacts gene regulation. By incorporating multiple optimal and near optimal solutions into this analysis, we provide evidence that the observed hierarchical structure persists not only across scales but across a variety of plausible high-scoring domain sets. However, multiple optimal solutions are not necessary to quantify the hierarchical structure of the domains since single optimal solutions across scales can already reveal a hierarchical structure. There are many more near-optimal solutions at higher values of *γ* since the domain sizes tend to be smaller. For this special case, it would be desirable to develop a method that more concisely characterizes these larger solution spaces, and this is an interesting direction for future work. The quantitative evidence of the hierarchical structure of topological domains also motivates the development of novel methods for domain discovery that directly account for such hierarchy in the models they assume and the functions they optimize.

The method for discovering topological domains that we have introduced is practical for existing datasets. Our implementation is able to compute the consensus set of domains for the human fibroblast cell line and extract the consensus set in 24 minutes when run on a personal computer with 2.3GHz Intel Core i5 processor and 8Gb of RAM. Computing optimal and near-optimal solutions adds only a small overhead to overall running time: when computing 20 top optimal and near-optimal solutions per each *γ* setting (with *γ* 0.0-0.9 with a step of 0.05) the computation finishes in 25 minutes 34 seconds.

A preliminary version of this manuscript appeared in the 2013 Workshop on Algorithms for Bioinformatics [25].

## Availability and requirements

A C++11 implementation of the algorithms and instructions for compilation and use are available at http://www.cs.cmu.edu/~ckingsf/software/armatus/.

## Competing interests

The authors declare that they have no competing interests.

## Authors’ contributions

DF, RP, and GD contributed equally to the development of methods and manuscript. CK contributed to the manuscript. All authors reviewed the manuscript and approved it.
